# Supplementary material for: Xylem Sap Mycobiota in Grapevine Naturally Infected with Xylella fastidiosa: A Case Study: Interaction of Xylella fastidiosa with Sclerotinia sclerotiorum
Source: Plants (Basel). 2025 Jun 27;14(13):1976. doi: 10.3390/plants14131976 (PMC12252444; doi:10.3390/plants14131976)
Supplement: Supplementary file 1 [file plants-14-01976-s001.zip › plants-3659204-supplementary.pdf]

Supplementary Table S1. Morphological traits used for the fungal characterization of the studied genera and fungal complexes shown in Figure 1.

	Key Diagnostic Features	
**Fungal Group**	**Macroscopic Traits (Culture)**	**Microscopic Traits**
***Phoma* complex**	Slow-growing colonies, grayish to olivaceous, sometimes producing pinkish pigment.	Pycnidia with ostiole, globose to flask-shaped, producing slim conidial masses; hyaline, aseptate conidia.
***Phomopsis* / *Diaporthe* complex**	Creamy to gray colonies, often with concentric zonation or black pycnidia.	Two conidial types; Alpha conidia: ellipsoid, aseptate; Beta conidia: filiform, curved or straight.
Alternaria alternata	Fast-growing, dark olive to blackish colonies, often with zonation.	Conidia large (20–63 µm long × 7–18 µm wide) with apical beak, muriform (both transverse and longitudinal septa), often in chains.; typical dark pigmentation.
***Cladosporium* spp.**	Velvety to powdery colonies, olive-gray to greenish-brown in color.	Conidia in branching chains, with scars (coronate hilum); conidiophores branched
Aureobasidium pullulans	Colonies initially yeast-like cells, turning dark and leathery with age.	Dimorphic nature; production of melanin; presence of both yeast and filamentous forms and dark-walled chlamydospores may be present. Budding blastoconidia produced in large numbers; hyaline, oval to ellipsoidal, unicellular; size: 4–10 × 2–4 µm.
Rhodotorula mucilaginosa	Yeast-like growth with bright pink to coral colonies; smooth, mucoid, shiny	Oval to round yeast cells, reproducing by budding; absence of pseudohyphae or mycelium.
***Penicillium* spp.**	Rapidly growing, velvety to powdery colonies, typically blue-green with a white margin.	Brush-like conidiophores with branched metulae and phialides; conidia produced in chains.
***Botryosphaeria* complex**	Dark pigmented, slow- to moderate-growing colonies.	Pycnidia or pseudothecia; fusiform, aseptate or septate conidia, occasionally with appendages. Asexual and sexual structures; dark, thick-walled pycnidia characteristic of the complex.

	Key Diagnostic Features	
**Fungal Group**	**Macroscopic Traits (Culture)**	**Microscopic Traits**
***Phaeoacremonium / Phaeomoniella* spp.**	Pale brown to dark pigmented colonies; often slow-growing.	Pigmented hyphae; slimy phialidic conidia; presence of type-specific phialides. phialidic conidiogenesis
***Aspergillus* spp.**	Powdery colonies, green or black depending on species; rapid growth.	Conidiophores terminate in vesicle; phialides in uniseriate or biseriate arrangements; conidia in chains.
**Yeast-like fungi (general)**	Creamy, smooth, shiny colonies; white to pink or tan in color colony pigmentation	Oval or ellipsoidal yeast cells; reproduce by budding;<br><br>pseudohyphae presence/absence
Sclerotinia sclerotiorum	Rapid growth; white surface, cottony to fluffy aerial mycelium that covers the entire plate surface and black spherical to elongated sclerotia of 6.4 (2 to 10) mm.	Numerous black, irregularly shaped sclerotia form on colony surface and within agar, variable in size (2–10 mm). Applanate or embedded in agar, often peripheral or scattered across the colony.<br><br>Lack of conidia, typically non-conidial; identification relies on mycelial and sclerotial traits.

Culture characteristics were assessed on PDA 2% medium or Sabouraud medium for 3-7 days in a growth chamber at 25°C, 12 h of light and 12 h of darkness. Microscopic structures were observed in slide cultures stained with lactophenol cotton blue.
